# Supplementary material for: Sea ice dynamics across the Mid-Pleistocene transition in the Bering Sea
Source: Nat Commun. 2018 Mar 5;9:941. doi: 10.1038/s41467-018-02845-5 (PMC5838228; doi:10.1038/s41467-018-02845-5)
Supplement: Supplementary file 1 — Supplementary Information [file 41467_2018_2845_MOESM1_ESM.pdf]

Supplementary Material

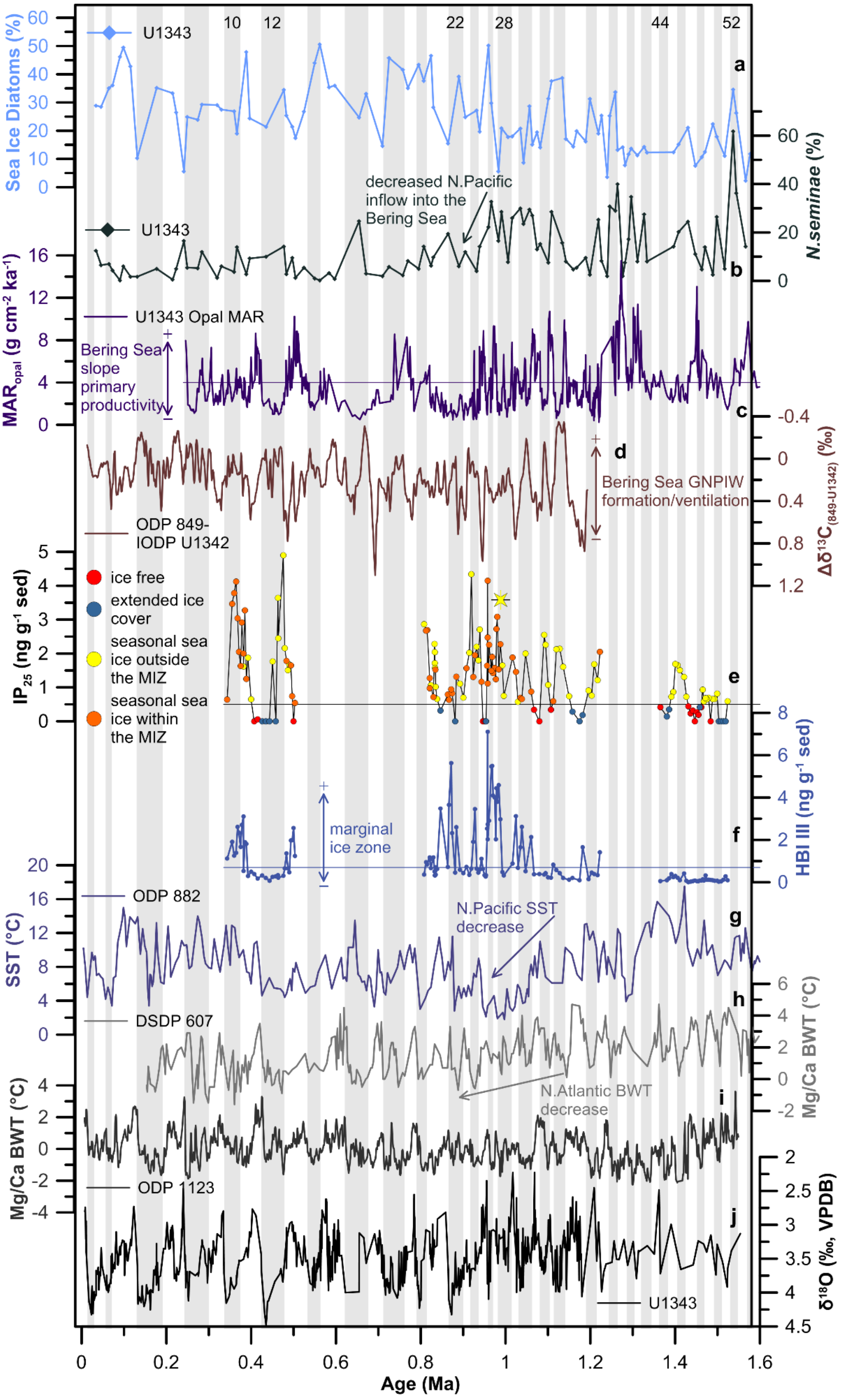

Supplementary Figure 1. Sea ice dynamics in the Bering Sea (colour coded sea ice states) in comparison with regional and global climate records.

Overview of regional and global climate records across the Pleistocene. (**a**) Abundance of sea ice diatoms in Site U1343[1] (light blue). (**b**) Abundance of *N.seminae* in sediments of Site U1343[1] (dark green). (**c**) Site U1343 mass accumulation rate of biogenic opal[2] (MAR_{opal}) (purple). (**d**) $\Delta\delta^{13}C_{(849\text{-}U1342)}$[3] indicative of GNPIW ventilation/formation (brown). (**e**) Site U1343 IP_{25} record (this study), sea ice regimes are colour-coded as determined in the manuscript (red: ice free, blue: extended ice cover, orange: seasonal sea ice within the MIZ, yellow: seasonal sea ice outside the MIZ). The IP_{25} peak labelled with an asterisk denotes the first glacial interval (MIS 28) characterised by a late-glacial/deglacial sea ice maximum. (**f**) Site U1343 HBI III (blue) record (this study). (**g**) Alkenone-based North Pacific SSTs ODP Site 882[4] (light purple). (**h**) BWT in DSDP Site 607[5] in the North Atlantic (light grey). (**i**) BWT in ODP Site 1123[6] in the southwest Pacific (dark grey). (**j**) Site U1343 $\delta^{18}O_b$ record (black)[7]. The horizontal lines in the MAR_{opal}, IP_{25}, and HBI III records indicate the boundary values for interpreting the sea ice states (as indicated in Table 2). Grey bars indicate glacial intervals, white bars represent interglacials (numbers at the top correspond to MIS, MIS boundaries from Lisiecki and Raymo[8]).

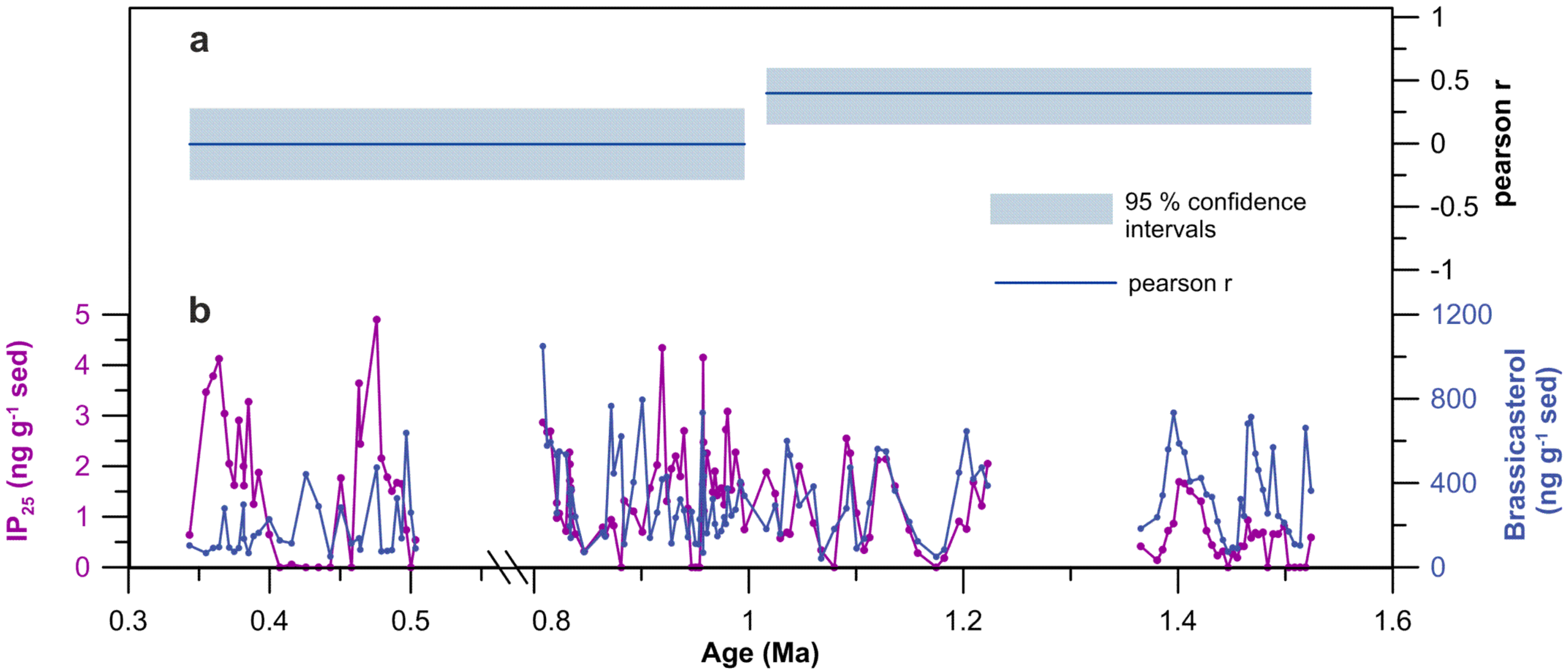

Supplementary Figure 2. Correlation of Brassicasterol and IP_{25} across all three measured intervals in Site U1343.

(**a**) The Pearson r correlation coefficient[9] calculated of IP_{25} (violet) and brassicasterol (blue). The interval has been divided into two subsections from 1.53-0.95 Ma and from 0.95-0.34 Ma. (**b**) Brassicasterol and IP_{25} across all three intervals 1.53-1.36 Ma, 1.22-0.8 Ma, 0.5-0.34 Ma measured in this study.

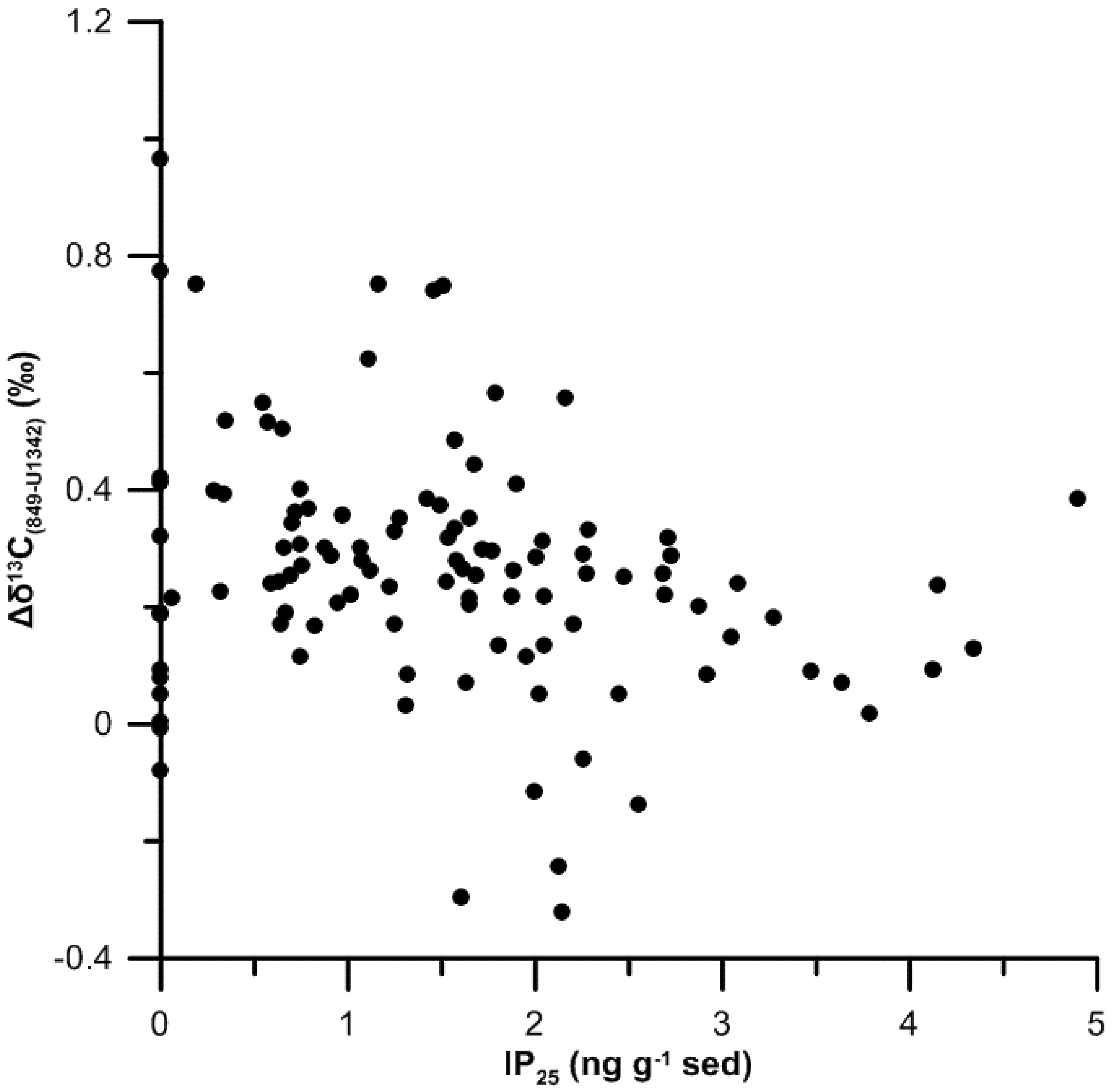

Supplementary Figure 3. Correlation of the sea ice proxy IP_{25} with Bering Sea intermediate water ventilation.

A cross-plot of IP_{25} from Site U1343 and $\Delta\delta^{13}C_{(849\text{-}U1342)}$[3] a proxy for intermediate water ventilation at Site U1342 (IP_{25}-$\Delta\delta^{13}C_{(849\text{-}U1342)}$: $r_{xy} = -0.255$ with 95% Student's t confidence intervals [-0.473; -0.009], n = 110). $\Delta\delta^{13}C_{(849\text{-}U1342)}$ has been re-sampled at the same age points as the U1343 IP_{25} record, using AnalySeries 2.0.8[10].

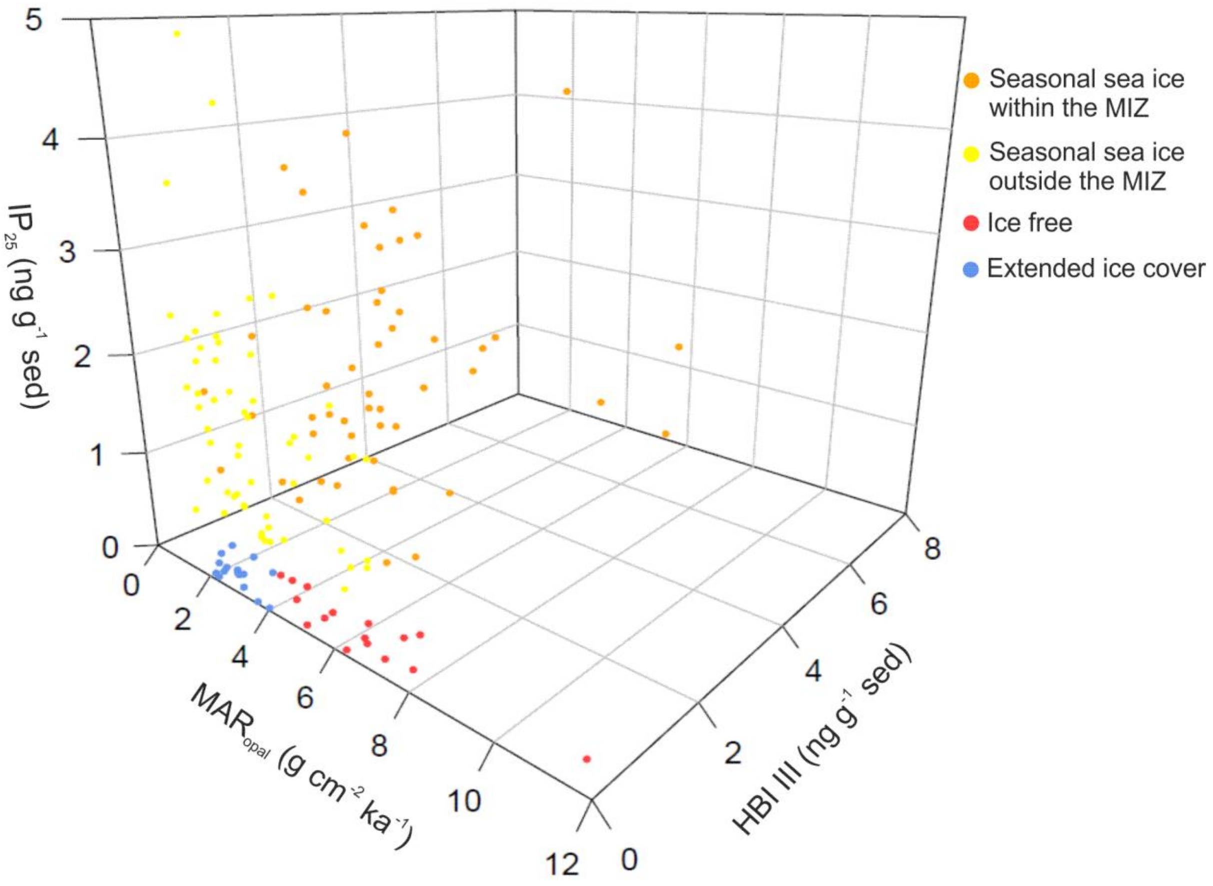

Supplementary Figure 4. Characterization of sea ice states at the eastern Bering Sea slope.

Data points are clustered according to their predominant sea ice state (for biomarker and MAR_{opal} boundaries see Table 2 in the main manuscript). Blue data points represent extended sea ice cover, red ice free conditions, orange seasonal sea ice within the MIZ, and yellow seasonal sea ice outside the MIZ. The ice free and extended ice cover scenarios are separated by MAR_{opal} of 4 g cm^{-2} ka^{-1}, determined from sea ice studies[11] and MAR_{opal} in site U1343 across Termination I (Supplementary Fig. 5).

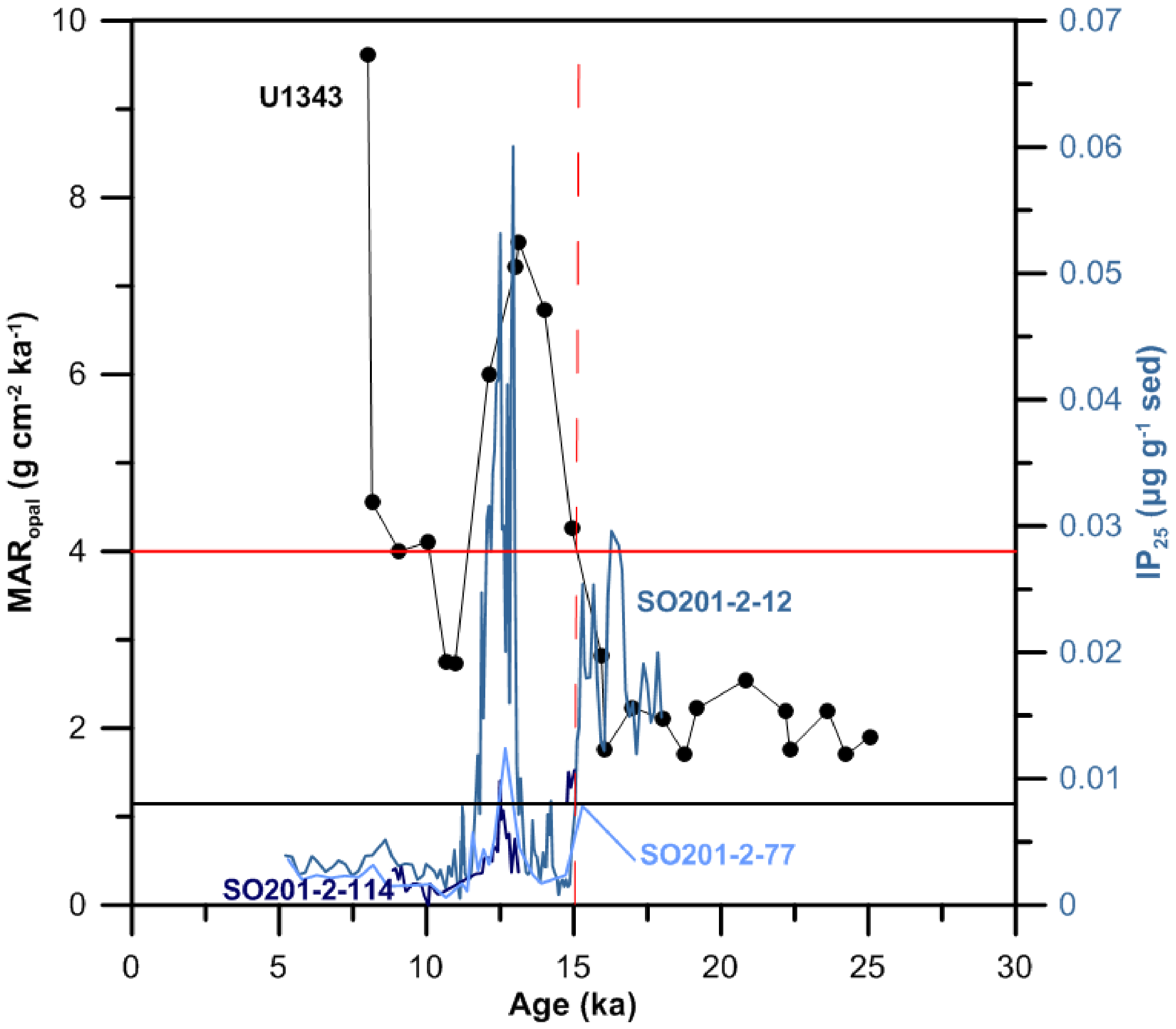

Supplementary Figure 5. Determination of MAR_{opal} under ice free and extended sea ice conditions using sea ice data in the eastern Bering Sea[11] and MAR_{opal} at Site U1343[2] across Termination I.

Termination I sea ice studies based on IP_{25} from three sediment cores in the western Bering Sea and the north west Pacific[11] (different shades of blue) and the mass accumulation rate of biogenic opal (MAR_{opal}) at Site U1343 (black). The black horizontal line represents IP_{25} values of 0.008 μg g^{-1} sed, the boundary between seasonal and ice free/extended sea ice conditions in the western Bering Sea, as identified by Meheust et al.[11]. Seasonal/ice free conditions are observed from 15 ka BP onwards (red dashed vertical line) in the western Bering Sea. At 15 ka MAR_{opal} in U1343 is at 4 g cm^{-2} ka^{-1} (red horizontal line), taken as the boundary to identify ice free versus extended sea ice conditions in Site U1343.

Supplementary References

1. Teraishi A, Suto I, Onodera J, Takahashi K. Diatom, silicoflagellate and ebridian biostratigraphy and paleoceanography in IODP 323 Hole U1343E at the Bering slope site. *Deep Sea Research Part II: Topical Studies in Oceanography* **125**, 18-28 (2016).

2. Kim S, Takahashi K, Khim B-K, Kanematsu Y, Asahi H, Ravelo AC. Biogenic opal production changes during the Mid-Pleistocene Transition in the Bering Sea (IODP Expedition 323 Site U1343). *Quaternary Research* **81**, 151-157 (2014).

3. Knudson KP, Ravelo AC. North Pacific Intermediate Water circulation enhanced by the closure of the Bering Strait. *Paleoceanography* **30**, 1287-1304 (2015).

4. Martínez-Garcia A, Rosell-Melé A, McClymont EL, Gersonde R, Haug GH. Subpolar Link to the Emergence of the Modern Equatorial Pacific Cold Tongue. *Science* **328**, 1550-1553 (2010).

5. Sosdian S, Rosenthal Y. Deep-Sea Temperature and Ice Volume Changes Across the Pliocene-Pleistocene Climate Transitions. *Science* **325**, 306-310 (2009).

6. Elderfield H*, et al.* Evolution of Ocean Temperature and Ice Volume Through the Mid-Pleistocene Climate Transition. *Science* **337**, 704-709 (2012).

7. Asahi H*, et al.* Orbital-scale benthic foraminiferal oxygen isotope stratigraphy at the northern Bering Sea Slope Site U1343 (IODP Expedition 323) and its Pleistocene paleoceanographic significance. *Deep Sea Research Part II: Topical Studies in Oceanography* **125–126**, 66-83 (2016).

8. Lisiecki LE, Raymo ME. A Pliocene-Pleistocene stack of 57 globally distributed $\delta^{18}O$ records. *Paleoceanography* **20**, PA1003 (2005).

9. Mudelsee M. Estimating Pearson's Correlation Coefficient With Bootstrap Confidence Interval From Serially Dependent Time Series. *Mathematical Geology* **35**, 651–665 (2003).

10. Paillard D, Labeyrie L, Yiou P. Macintosh Program performs time-series analysis. *Eos, Transactions American Geophysical Union* **77**, 379-379 (1996).

11. Méheust M, Stein R, Fahl K, Max L, Riethdorf J-R. High-resolution IP25-based reconstruction of sea-ice variability in the western North Pacific and Bering Sea during the past 18,000 years. *Geo-Marine Letters* **36**, 101-111 (2016).
